# Author Correction: Microbiome differential abundance methods produce different results across 38 datasets

**DOI:** 10.1038/s41467-022-28401-w

**Published:** 2022-02-03

**Authors:** Jacob T. Nearing, Gavin M. Douglas, Molly G. Hayes, Jocelyn MacDonald, Dhwani K. Desai, Nicole Allward, Casey M. A. Jones, Robyn J. Wright, Akhilesh S. Dhanani, André M. Comeau, Morgan G. I. Langille

**Affiliations:** 1 Department of Microbiology and Immunology, Dalhousie University, Halifax, NS Canada; 2 Department of Mathematics and Statistics, Dalhousie University, Halifax, NS Canada; 3 Department of Computer Science, Dalhousie University, Halifax, NS Canada; 4 Integrated Microbiome Resource, Dalhousie University, Halifax, NS Canada; 5 Department of Civil and Resource Engineering, Dalhousie University, Halifax, NS Canada; 6 Department of Pharmacology, Dalhousie University, Halifax, NS Canada

**Keywords:** Data processing, High-throughput screening, Statistical methods, Microbiome

Correction to: *Nature Communications* 10.1038/s41467-022-28034-z, published online 17 January 2022.

In the original version of this Article, the reference “Turnbaugh, P. J. et al. A core gut microbiome in obese and lean twins. *Nature* **457**, 480–484 (2009)” was duplicated labelled as 59 and 80 in the Reference section. The duplicate reference has now been deleted and the following references 81, 82, and 83 have been changed to 80, 81, and 82, respectively. In addition, the relevant citations in the text have been corrected. These have been corrected in both the PDF and HTML versions of the Article.

