# Supplementary material for: Microbiome differential abundance methods produce different results across 38 datasets
Source: Nat Commun. 2022 Jan 17;13:342. doi: 10.1038/s41467-022-28034-z (PMC8763921; doi:10.1038/s41467-022-28034-z)
Supplement: Supplementary file 1 — Supplementary Information [file 41467_2022_28034_MOESM1_ESM.pdf]

Microbiome differential abundance methods produce different results across 38 datasets

Nearing *et al.*

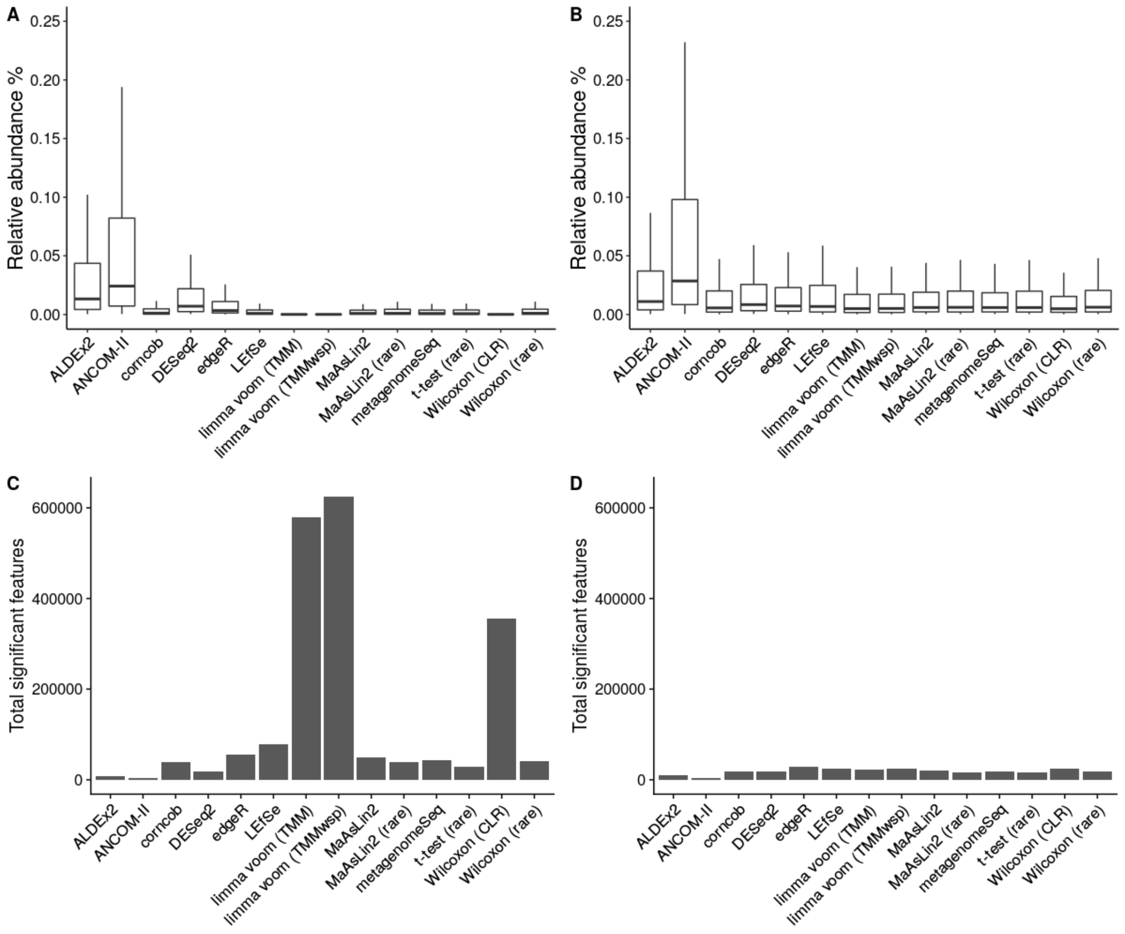

Supplementary Figure 1. Counts and relative abundances of significant features by tool across all 38 datasets. (A and B) Boxplots of relative abundance per significant features for the (A) unfiltered and (B) prevalence-filtered approaches over the 38 datasets defined in supplemental table 1. Interquartile range (IQR) of boxplots represent the 25th and 75th percentiles while maxima and minima represent the maximum and minimum values outside 1.5 times the IQR. Notch in the middle of the boxplot represent the median. (C and D) Total number of significant features for the (C) unfiltered and (D) prevalence-filtered approaches summed over the 38 datasets defined in supplemental table 1. Source data are provided as a Source Data file.

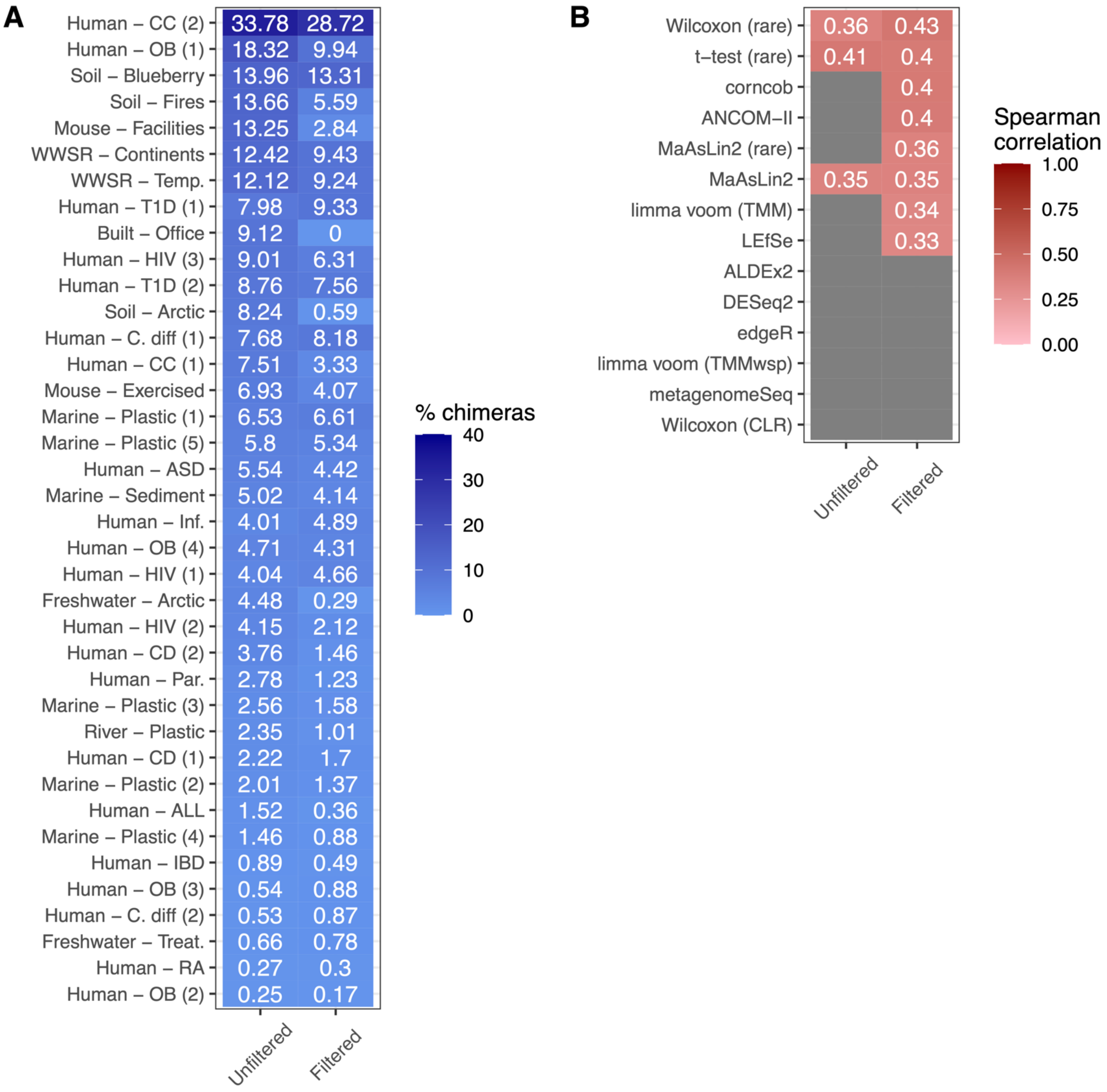

Supplementary Figure 2. Prevalence of chimeric amplicon sequence variants (as inferred through a reference-based approach) varies across the study datasets. (A) Percentage of amplicon sequence variants (ASVs), in the non-rarefied datasets. (B) Significant Spearman correlation coefficients between the percent chimeras and the percent significant ASVs across the datasets. Inferences of chimeric ASVs are expected to be enriched for false positives when reference-based chimera checking approaches are applied, which complicates how to interpret these observations. Source data are provided as a Source Data file.

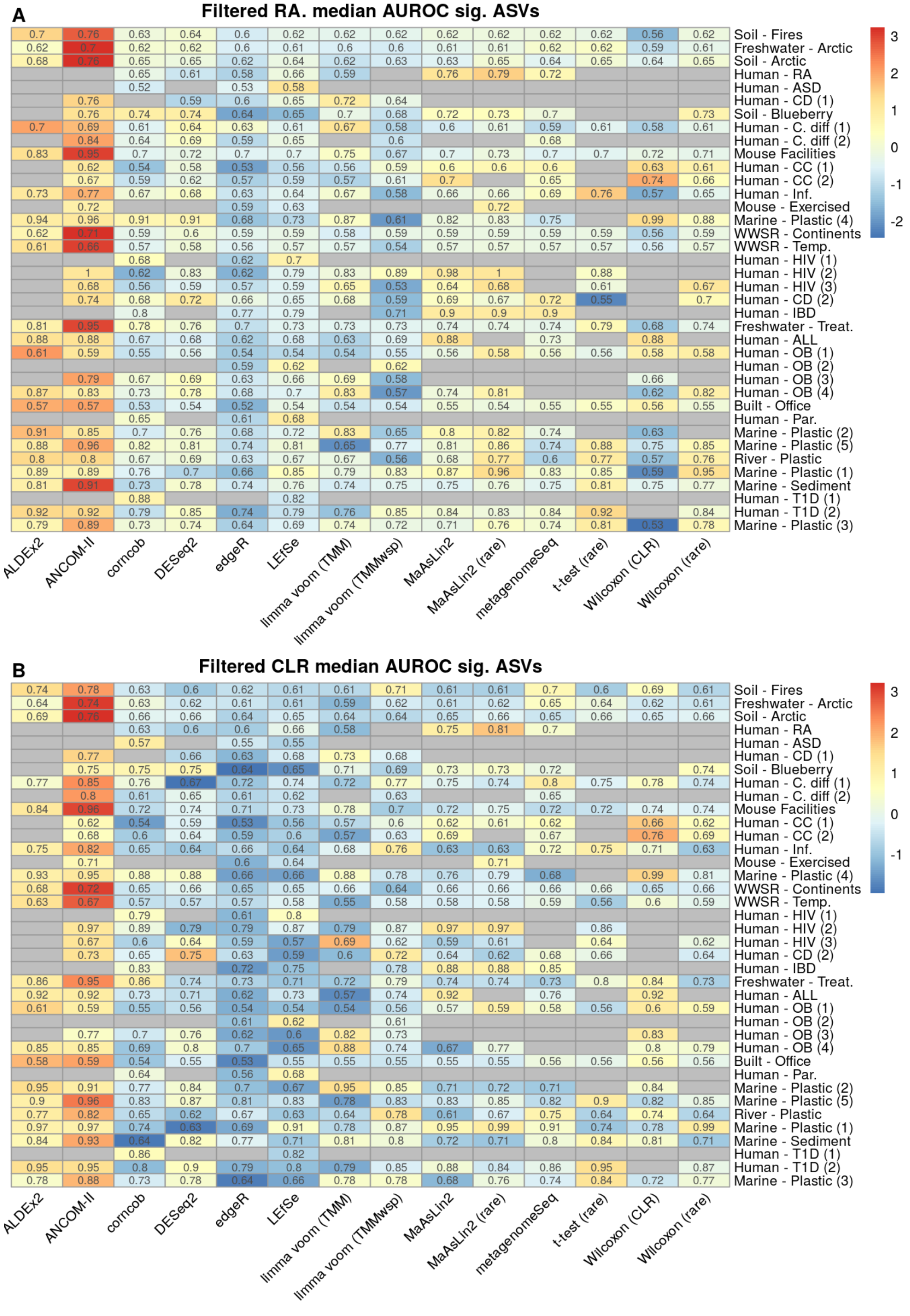

Supplemental Figure 3. Heatmap of mean area under the receiver operator curve (AUROC) of significant amplicon sequence variants (ASVs) identified by each tool. The mean AUROC was calculated from the AUROC of each individual ASV that was identified as being differentially abundant by each tool per dataset. AUROC was calculated for each ASV using both relative abundances (A) and center-log-ratio abundance (B). Only the results of the significant hits identified in the filtered dataset are shown. The heatmap colours indicate the scaled and mean-centred mean values by dataset. Source data are provided as a Source Data file.

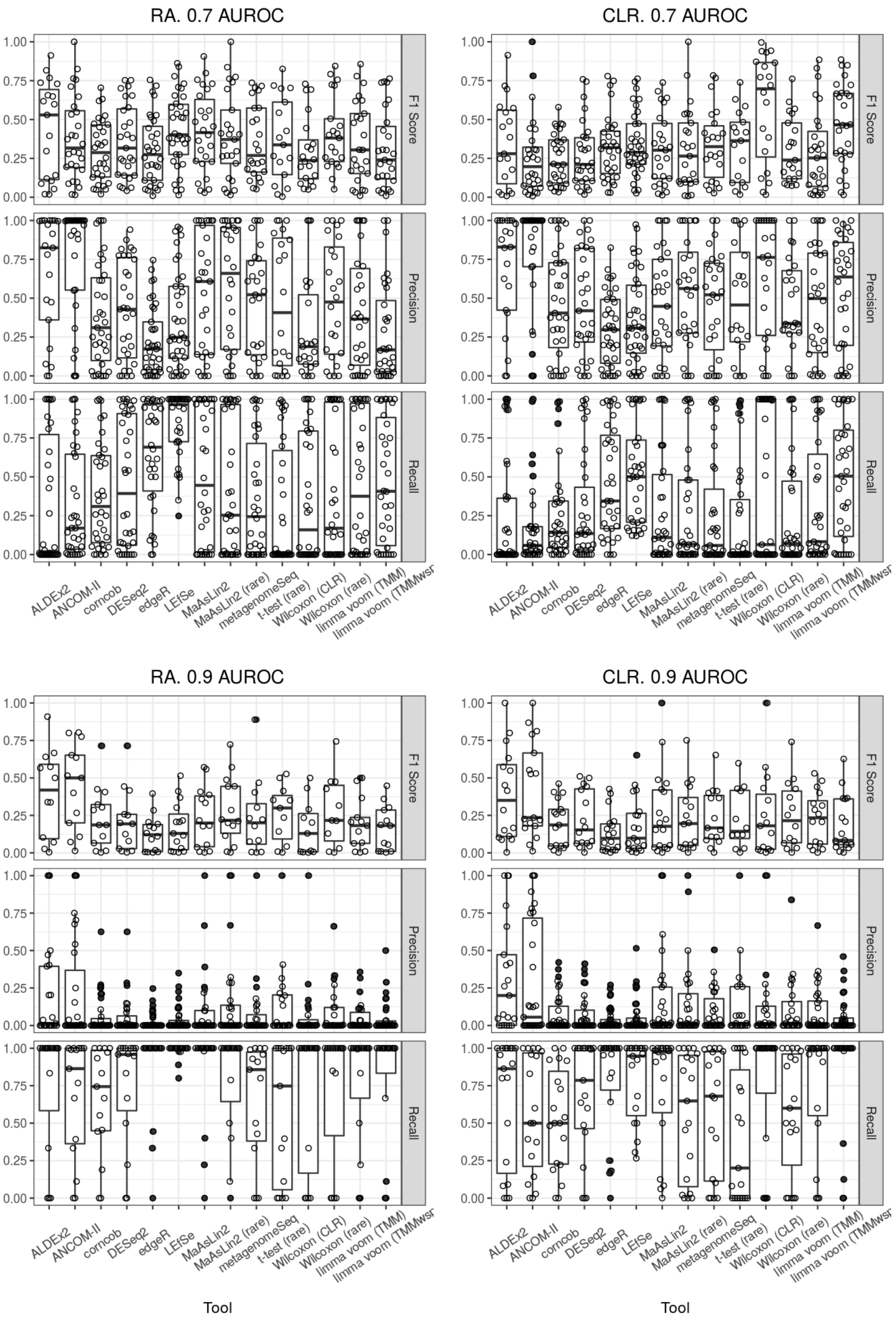

Supplemental Figure 4. Precision, Recall and F1 score of identified ASVs above specific AUROC cutoffs. The AUROC for each ASV within a dataset was calculated to identify features that were discriminatory between metadata groupings (i.e., that can accurately separate the samples into the correct groups). ASVs that reached an AUROC of 0.7 or 0.9 were then considered ASVs of importance for the calculation of the precision recall and F1 score for each differential abundance tool. AUROC for each ASV in every dataset was calculated using both relative abundances (from non-rarified tables) and centered log-ratio abundances. All 38 datasets (n=38) included in supplemental table 1 was included in this analysis. Interquartile range (IQR) of boxplots represent the 25th and 7th percentiles while maxima and minima represent the maximum and minimum values outside 1.5 times the IQR. Notch in the middle of the boxplot represent the median. Source data are provided as a Source Data file.

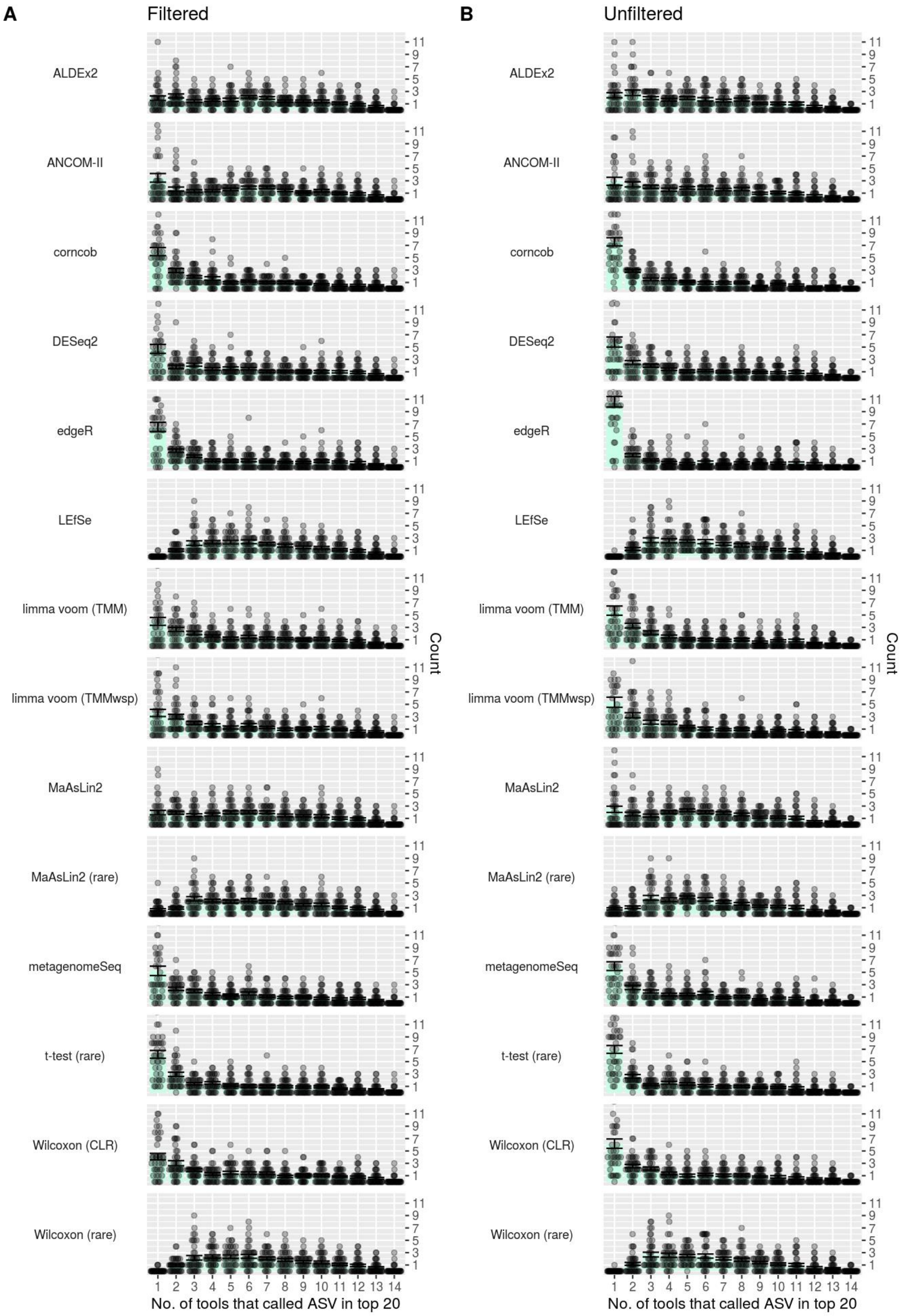

Supplemental Figure 5. Overlap between top 20 amplicon sequence variants (ASVs) identified by each differential abundance method. The top 20 ASVs identified by each differential abundance method were identified by ranking ASVs by the lowest significance value (except for ANCOM-II where the highest W statistic was used for ASV ranking). The number of tools that identified each of these ASVs were determined along with their frequencies for each tool. This was done using the results from running each differential abundance method on both filtered (A) and unfiltered (B) datasets. Bars represent the mean frequencies across all 38 datasets (n=38) and error bars represent the standard error of those means. Dots represent values from each individual dataset. Source data are provided as a Source Data file.

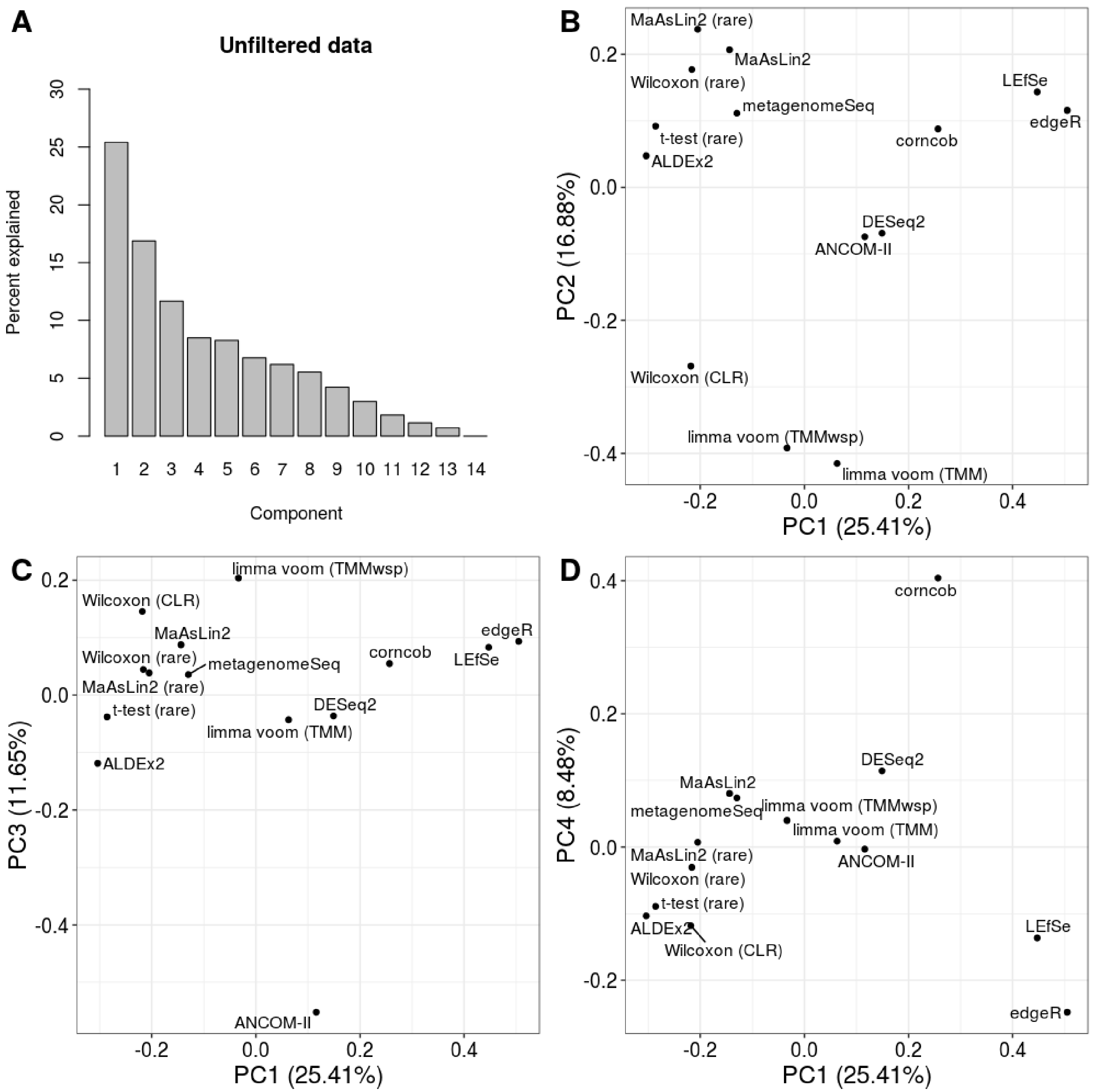

Supplementary Figure 6. Principal Coordinates Analysis on significant sets of amplicon sequence variants (based on unfiltered data). (A) Percentage explained by each component of the Principal Coordinates Analysis (PCoA). (B-D) Two-dimension summaries of PCoA as in Figure 3, but panels C and D visualize components three and four against the first component. Source data are provided as a Source Data file.

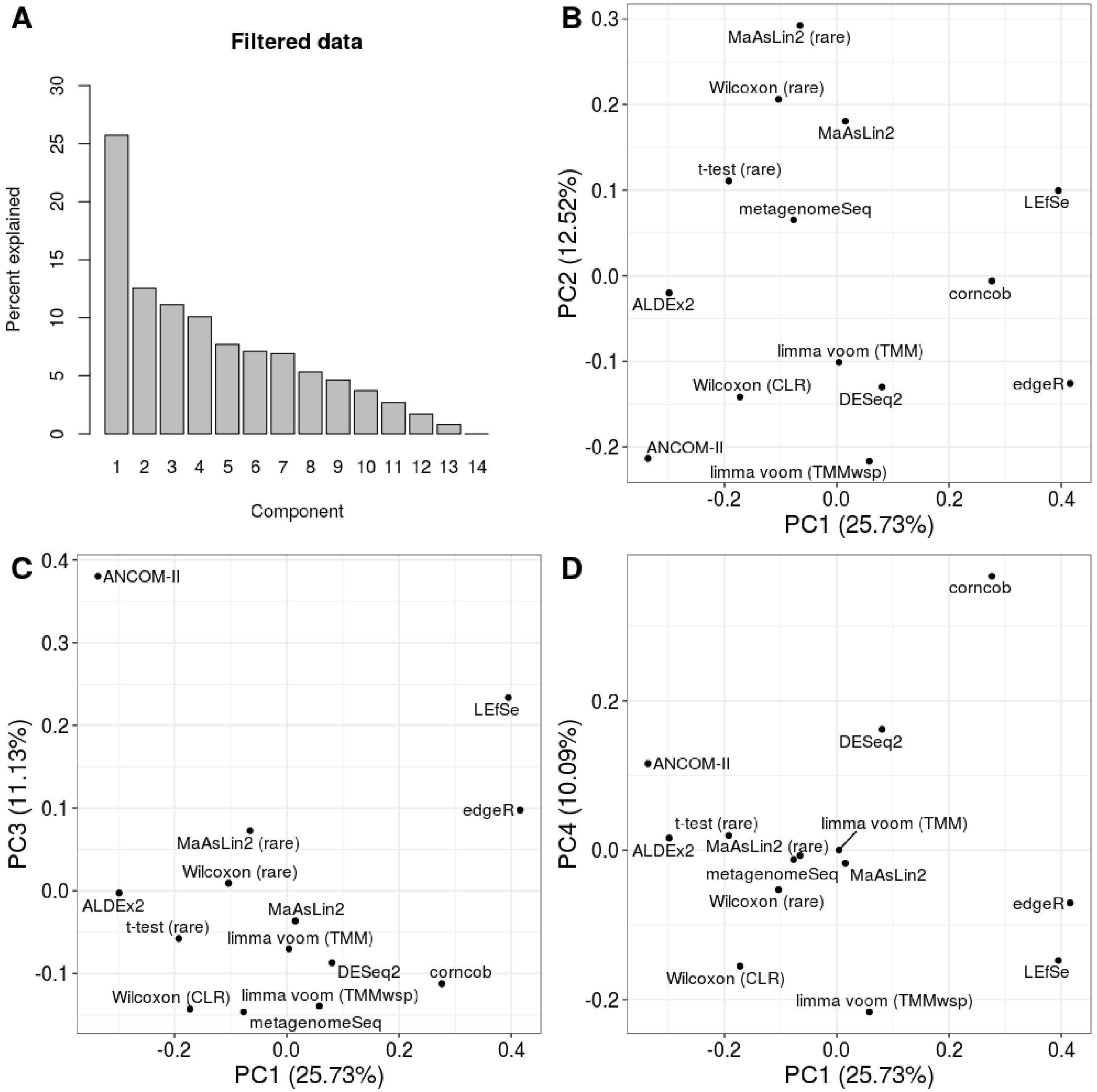

Supplementary Figure 7. Principal Coordinates Analysis on significant sets of amplicon sequence variants (based on prevalence filtered data). (A) Percentage explained by each component of the Principal Coordinates Analysis (PCoA). (B-D) Two-dimensional summaries of PCoA as in Figure 3, but panels C and D visualize components three and four against the first component. Source data are provided as a Source Data file.

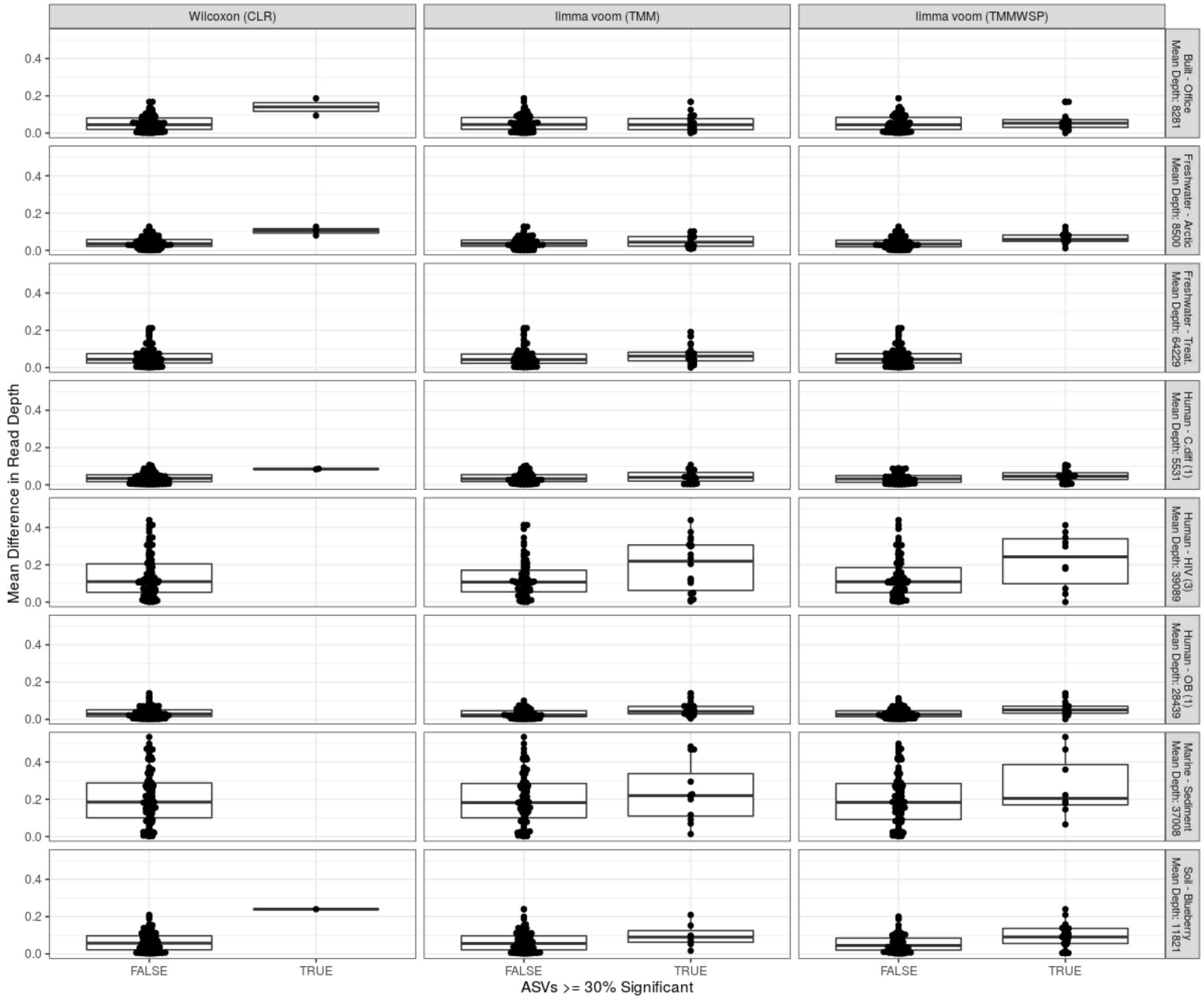

Supplemental Figure 8. Boxplot comparing mean read depth differences for replicates in unfiltered analysis that resulted in 30% or more ASVs being identified as significant during the false positive analysis. For each dataset and replicate in our false positive analysis we checked whether there was a difference in mean read depth between the two tested groups for the Wilcoxon (CLR), limma voom (TMM), and limma voom (TMMWSP). These tools were chosen due to their inconsistent findings across replicates, in some cases identifying greater than 90% of amplicon sequence variants (ASVs) as being differentially abundant. The y-axis corresponds to the difference between the group mean read depths normalized by the mean read depth of all samples. On the x-axis, the FALSE category corresponds to replicates where fewer than 30% of ASVs were significant, while TRUE represents replicates where at least 30% or more of the tested ASVs were significant. A total of eight unfiltered datasets were included in this analysis resulting in 800 data points per tool representing 100 replicates from each dataset. Interquartile range (IQR) of boxplots represent the 25th and 7th percentiles while maxima and minima represent the maximum and minimum values outside 1.5 times the IQR. Notch in the middle of the boxplot represent the median. Source data are provided as a Source Data file.

Supplementary Table 1. Consistency of significant genera calls across obesity datasets

Tool	No. sig. genera	Max overlap	Mean exp.	Mean obs.	Fold diff.	*p*
MaAsLin2 (rare)	8	2	1.019	1.25	1.227	0.003
MaAsLin2	20	3	1.071	1.25	1.167	0.004
t-test (rare)	6	2	1.012	1.167	1.153	0.015
ALDEx2	24	3	1.064	1.167	1.097	0.025
limma voom (TMMwsp)	34	3	1.127	1.235	1.096	0.036
corncob	34	3	1.136	1.206	1.062	0.135
LEfSe	51	3	1.215	1.275	1.049	0.151
limma voom (TMM)	44	3	1.164	1.205	1.035	0.207
Wilcoxon (rare)	22	2	1.062	1.091	1.027	0.249
edgeR	68	3	1.327	1.338	1.008	0.415
Wilcoxon (CLR)	47	3	1.167	1.17	1.003	0.449
DESeq2	40	2	1.123	1.125	1.002	0.465
metagenomeSeq	4	1	1.009	1	0.991	0.028
ANCOM-II	8	1	1.015	1	0.985	0.105

No. sig. genera: Number of genera significant in at least one dataset;
Max overlap: Maximum number of datasets where a genus was called by significant by this tool;
Mean exp.: Mean number of datasets that each genus is expected to be significant in (of the genera that are significant at least once);
Mean obs.: Mean number of datasets that each genus was observed to be significant in (of the genera that are significant at least once);
Fold diff.: Fold difference of mean observed over mean expected number of times significant genera are found across multiple datasets;
p: *p*-value based on one-tailed permutation test that used the 'Mean obs.' as the test statistic.
Source data are provided as a Source Data file.
