# Supplementary material for: Microbiome differential abundance methods produce different results across 38 datasets
Source: Nat Commun. 2022 Jan 17;13:342. doi: 10.1038/s41467-022-28034-z (PMC8763921; doi:10.1038/s41467-022-28034-z)
Supplement: Supplementary file 3 — Description of Additional Supplementary Files [file 41467_2022_28034_MOESM3_ESM.pdf]

Description of Additional Supplementary Files

File Name: Supplementary Data 1
Description: Contains all data to reproduce figures and tables shown within this manuscript with the exception of supplemental figure 1AB. Data to reproduce this figure can be found in the Github repository referenced in the data availablity section.
