# Supplementary material for: Microbiome differential abundance methods produce different results across 38 datasets
Source: Nat Commun. 2022 Jan 17;13:342. doi: 10.1038/s41467-022-28034-z (PMC8763921; doi:10.1038/s41467-022-28034-z)
Supplement: Supplementary file 5 — Reporting Summary [file 41467_2022_28034_MOESM5_ESM.pdf]

nature portfolio

Corresponding author(s): Jacob Nearing

Last updated by author(s): Dec 10th, 2021

Reporting Summary

Nature Portfolio wishes to improve the reproducibility of the work that we publish. This form provides structure for consistency and transparency in reporting. For further information on Nature Portfolio policies, see our Editorial Policies and the Editorial Policy Checklist.

Statistics

For all statistical analyses, confirm that the following items are present in the figure legend, table legend, main text, or Methods section.

n/a	Confirmed	
☐	☒	The exact sample size (*n*) for each experimental group/condition, given as a discrete number and unit of measurement
☐	☒	A statement on whether measurements were taken from distinct samples or whether the same sample was measured repeatedly
☐	☒	The statistical test(s) used AND whether they are one- or two-sided<br>*Only common tests should be described solely by name; describe more complex techniques in the Methods section.*
☐	☒	A description of all covariates tested
☐	☒	A description of any assumptions or corrections, such as tests of normality and adjustment for multiple comparisons
☐	☒	A full description of the statistical parameters including central tendency (e.g. means) or other basic estimates (e.g. regression coefficient) AND variation (e.g. standard deviation) or associated estimates of uncertainty (e.g. confidence intervals)
☐	☒	For null hypothesis testing, the test statistic (e.g. *F*, *t*, *r*) with confidence intervals, effect sizes, degrees of freedom and *P* value noted<br>*Give P values as exact values whenever suitable.*
☒	☐	For Bayesian analysis, information on the choice of priors and Markov chain Monte Carlo settings
☒	☐	For hierarchical and complex designs, identification of the appropriate level for tests and full reporting of outcomes
☐	☒	Estimates of effect sizes (e.g. Cohen's *d*, Pearson's *r*), indicating how they were calculated

Our web collection on statistics for biologists contains articles on many of the points above.

Software and code

Policy information about availability of computer code

Data collection	Datasets were collected from a number of different dataset repositories using secure file transfer protocol (sftp) or downloading via wget or links depending on data source.
Data analysis	Raw 16S rRNA gene sequencing datasets were processed using QIIME2 (version 2019.7) and its included packages Deblur (version 2019.7) , and VSEARCH (version 2019.7). All differential abundance tools examined along with their tested version numbers are listed in table 1. All R packages used for additional analysis are listed within the linked GitHub repository under the data availability statement.<br><br>These packages include:<br>"GUniFrac" Version: 1.1<br>"ALDEx2" Version: 1.18.0<br>"exactRankTests" Version: 0.8.31<br>"nlme" Version: 3.1.149<br>"dplyr" Version: 0.8.5<br>"ggplot2" Version: 3.3.0<br>"compositions" Version: 1.40.2<br>"corncob" Version: 0.1.0<br>"phyloseq" Version: 1.29.0<br>"DESeq2" Version: 1.26.0<br>"edgeR" Version: 3.28.1<br>"limma" Version: 3.42.2<br>"Maaslin2" Version: 0.99.12<br>"metagenomeSeq" Version: 1.28.2<br>"corrplot" Version: 0.85<br>"pheatmap" Version: 1.0.12

March 2021

"gridExtra" Version: 2.3
"cowplot" Version: 1.0.0
"ggrepel" Version: 0.8.1
"doParallel" Version: 1.0.15
"doMC" Version: 1.3.5
"matrixStats" Version: 0.56.0
"reshape2" Version: 1.4.4
"plyr" Version: 1.8.6
"ggplotify" Version: 0.0.5
"ggbeeswarm" Version: 0.6.0
"scales" Version: 1.1.0
"tidyverse" Version: 1.3.0
"vegan" Version: 2.5.6
"parallelDist" Version: 0.2.4

For manuscripts utilizing custom algorithms or software that are central to the research but not yet described in published literature, software must be made available to editors and reviewers. We strongly encourage code deposition in a community repository (e.g. GitHub). See the Nature Portfolio guidelines for submitting code & software for further information.

Data

Policy information about availability of data

All manuscripts must include a data availability statement. This statement should provide the following information, where applicable:

- Accession codes, unique identifiers, or web links for publicly available datasets
- A description of any restrictions on data availability
- For clinical datasets or third party data, please ensure that the statement adheres to our policy

All data that was used to generate any main figures or supplemental figures are available as source data as either excel documents or for large datasets csv files. This data is available at https://github.com/nearinj/Comparison_of_DA_microbiome_methods.
The processed datasets and metadata files are available at https://figshare.com/articles/dataset/16S_rRNA_Microbiome_Datasets/14531724. The accessions and/or locations of the raw data for each tested dataset are listed in Supplementary Data 1. SILVA database v138 is available at: https://www.arb-silva.de/documentation/release-138/.

Field-specific reporting

Please select the one below that is the best fit for your research. If you are not sure, read the appropriate sections before making your selection.

☒ Life sciences ☐ Behavioural & social sciences ☐ Ecological, evolutionary & environmental sciences

For a reference copy of the document with all sections, see nature.com/documents/nr-reporting-summary-flat.pdf

Life sciences study design

All studies must disclose on these points even when the disclosure is negative.

Sample size	Sample sizes were based on previously published openly shared datasets found in various sources including the microbiomeHD database (Duvallet et al., 2017), in house publicly available datasets sequenced by the Integrated Microbiome Resource (imr.bio), processed datasets available on Qiita (Gonzalez et al., 2018) or datasets available on NCBI (NCBI Resource Coordinators, 2018). The total number of datasets tested was determined based on the ability to test each tool on a wide range of different microbiome environments and data count distributions. This includes host associated, built and naturally occurring environments.
Data exclusions	All datasets that were examined within Duvallet et al. 2017, Meta-analysis of gut microbiome studies identifies disease-specific and shared responses, were included with the exception of a single obesity dataset (Zupancic et al. 2012, Analysis of the gut microbiota in the old order Amish and its relation to the metabolic syndrome) due to its high sparsity resulting in all OTUs being removed during our 10% prevalence analysis.
Replication	We have re-run our custom code a number of times to confirm the reproducibility of the results. Furthermore, we found that using 10 and 100 randomizations for sample labeling during false positive analysis was sufficient to create reproducibility results for each respective tool
Randomization	To evaluate the false positive rates of each DA method, eight datasets were selected for analysis based on having the largest sample sizes, while also being from diverse environment types. In each dataset, only the most frequent sample group was chosen for analysis to help ensure similar composition among samples tested. Within this grouping, random labels of either case or control were assigned to samples and the various differential abundance methods were tested on them. This was replicated 100 times for each dataset and tool combination aside from ALDEx2, ANCOM-II, and Corncob. These were run using 100 replicates in only 3 of the 8 datasets (Freshwater – Arctic, Soil – Blueberry, Human - OB (1)) with 100 ALDEx2 replications also being run in the Human - HIV (3) dataset. This was due to the long computational time required to run these tools on all datasets.<br><br>During the generation of rarified datasets a random seed was used to ensure reproducibility of these tables between pipeline runs.

Blinding	Blinding was not applicable in this study as we were comparing the capabilities of tools on the same datasets using the same alpha values with the exception of ANCOM-II which we used an internal significance cut-off of 0.9 which was determined before we began conducting any analysis.

Reporting for specific materials, systems and methods

We require information from authors about some types of materials, experimental systems and methods used in many studies. Here, indicate whether each material, system or method listed is relevant to your study. If you are not sure if a list item applies to your research, read the appropriate section before selecting a response.

Materials & experimental systems

n/a	Involved in the study
☒	☐ Antibodies
☒	☐ Eukaryotic cell lines
☒	☐ Palaeontology and archaeology
☒	☐ Animals and other organisms
☒	☐ Human research participants
☒	☐ Clinical data
☒	☐ Dual use research of concern

Methods

n/a	Involved in the study
☒	☐ ChIP-seq
☒	☐ Flow cytometry
☒	☐ MRI-based neuroimaging
